# The Burden of Rabies in Tanzania and Its Impact on Local Communities

**DOI:** 10.1371/journal.pntd.0002510

**Published:** 2013-11-07

**Authors:** Maganga Sambo, Sarah Cleaveland, Heather Ferguson, Tiziana Lembo, Cleophas Simon, Honorati Urassa, Katie Hampson

**Affiliations:** 1 Boyd Orr Centre for Population and Ecosystem Health, College of Medical, Veterinary and Life Sciences, University of Glasgow, Glasgow, United Kingdom; 2 Ifakara Health Institute, Ifakara, Morogoro, Tanzania; 3 Temeke Municipal Council, Livestock Office, Dar Es Salaam, Tanzania; The Global Alliance for Rabies Control, United States of America

## Abstract

**Background:**

Rabies remains a major public health threat in many parts of the world and is responsible for an estimated 55,000 human deaths annually. The burden of rabies is estimated to be around US$20 million in Africa, with the highest financial expenditure being the cost of post-exposure prophylaxis (PEP). However, these calculations may be substantial underestimates because the costs to households of coping with endemic rabies have not been investigated. We therefore aimed to estimate the household costs, health-seeking behaviour, coping strategies, and outcomes of exposure to rabies in rural and urban communities in Tanzania.

**Methods and Findings:**

Extensive investigative interviews were used to estimate the incidence of human deaths and bite exposures. Questionnaires with bite victims and their families were used to investigate health-seeking behaviour and costs (medical and non-medical costs) associated with exposure to rabies. We calculated that an average patient in rural Tanzania, where most people live on less than US$1 per day, would need to spend over US$100 to complete WHO recommended PEP schedules. High costs and frequent shortages of PEP led to poor compliance with PEP regimens, delays in presentation to health facilities, and increased risk of death.

**Conclusion:**

The true costs of obtaining PEP were twice as high as those previously reported from Africa and should be considered in re-evaluations of the burden of rabies.

## Author Summary

Rabies remains a major public health problem, although the means to control and prevent this disease are available through mass dog vaccination and provision of post-exposure prophylaxis (PEP) to people exposed to bites by rabid or suspect rabid animals. Despite its necessity as a life-saving measure to prevent the fatal onset of rabies, access to PEP is a major problem in developing countries. We used extensive investigative interviews to estimate rabies incidence (deaths and exposures) and questionnaires to bite victims and their families to investigate health-seeking behaviour and costs associated with receiving PEP, in four districts covering both rural and urban Tanzania. Frequent shortages at health centres limited prompt access to PEP. Suspect bite victims often had to travel long distances to major hospitals to receive costly PEP, causing delays and increasing the risk of developing rabies. We calculated that an average patient in rural Tanzania would need to spend over $100 to complete the WHO recommended PEP schedules, unaffordable for many Tanzanians, who survive under the poverty line. Our data shows that rabies imposes a disproportionate financial hardship and high risk of dying of rabies to rural poor families and highlights the need to re-evaluate the burden of rabies in Africa.

## Introduction

Rabies is a fatal zoonotic infection of the central nervous system caused by a *lyssavirus* [1]. This disease, which can affect all mammals, is transmitted in the saliva of infectious animals [2]. Rabies is endemic in low income countries, causing an estimated 55,000 human deaths each year with over 98% of these deaths following bites from rabid dogs [3]. Annual expenditure for rabies control and prevention in Asia and Africa has been estimated to exceed US$500 million, with most of these costs spent on provision of post-exposure prophylaxis (PEP) [3]. However, reliable data on disease burden are scarce in most low income countries, particularly across Africa [4], [5]. Rabies can be controlled through mass dog vaccination [6]–[10] and human deaths prevented through timely and appropriate PEP, which consists of rapid and thorough washing of the wound, completion of post-exposure vaccination schedules plus inoculation with rabies immunoglobulin (RIG) for severely exposed bite-victims [11]. Various PEP schedules are recommended by WHO and by the US Advisory Committee on Immunization Practices (ACIP), requiring different numbers of doses [12], [13]. Despite the effectiveness of PEP, many thousands of people still die from rabies especially in rural areas of Asia and Africa where canine rabies is endemic. These deaths are affected by the accessibility and affordability of PEP, which are key factors in determining human disease risk [14].

Access to health services has been defined as a multidimensional process that, in addition to the quality of care, involves geographical accessibility, availability of the right type of care for those who need it, financial accessibility, and acceptability of services [15]. Geographical factors are known to be important but are often overlooked when quantifying the economic burden of disease [16]–[21]. More generally, problems of access to adequate and appropriate health care are common in developing countries [22]–[26]. In most countries in Sub-Saharan Africa, there are many reasons why prompt access to appropriate PEP remains a serious challenge for bite-victims. Cold chain requirements for storage of vaccines between 2–8°C limit access to areas where electricity is available [27]. Frequent shortages of biologicals (vaccines and immunoglobulin), particularly in rural clinics also limits access [5], and WHO-recommended schedules that include administration of RIG are very rarely followed in Africa [3].

Tanzania is a low income country where canine rabies is endemic, with around 1,500 human rabies deaths estimated to occur annually [4]. Although some data are available on the treatment-seeking behaviour of bite victims and the associated costs of accessing rabies PEP in rural areas of northern Tanzania [5], this study presents a more complete evaluation of PEP costs and determinants of human disease risk, capturing a broad range of socioeconomic settings and including both urban and rural communities. This study provides detailed insight into the consequences of rabies exposures, by investigating the health-seeking behaviour and coping strategies of bite victims.

## Materials and Methods

### Study areas

**Figure 1:**
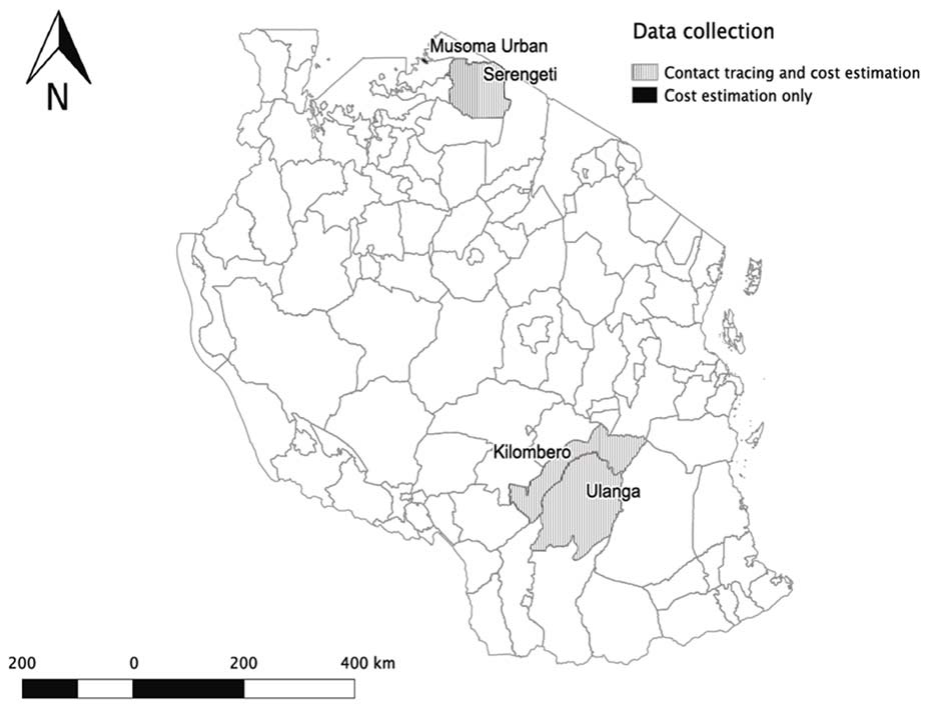
Map of Tanzania showing the study districts where incidences of rabies and cost data were collected. Gray shading indicates where extensive investigative interviews were administered and black shading where only cost data were collected.

The study was conducted in 4 districts in Tanzania, Serengeti and Musoma Urban in northern Tanzania and Ulanga and Kilombero in southern Tanzania, covering both urban and rural populations and the main livelihoods of agro-pastoralism and subsistence agriculture (Figure 1). Serengeti district is inhabited mainly by agro-pastoralists. The district hospital is located in Mugumu town, the headquarters of the district. There are no tarmac roads in Serengeti district and thus most roads are in poor condition especially during the rainy seasons. Musoma Urban district is located on the shores of Lake Victoria. In this district most people are employed in small business, small-scale agriculture, the Tanzanian civil service and fishing [28]. Musoma Urban is an urban district with better physical and social infrastructure and where all major town roads are tarmac. Musoma Urban serves as the headquarters of the Mara region and is where Mara regional hospital is located. In southern Tanzania, Ulanga and Kilombero districts are situated in the floodplain of the Kilombero valley. The majority of people living in these two districts are subsistence farmers, fishermen or pastoralists. District hospitals in Ulanga and Kilombero are located in Mahenge town and Ifakara town, which are the respective district headquarters. Both districts have very poor roads although there are a few short stretches of tarmac in the district capitals and on the steepest roads. Flooding in the Kilombero valley makes roads inaccessible during the rainy season, reducing access to health facilities.

We used the criteria of the Tanzanian National Bureau of Statistics (NBS) in differentiating rural and urban areas [28]. The definition of urban areas according to NBS, applied in this study, is that urban areas are regional and district headquarters with boundaries as identified by the Tanzanian Village Act of 1975 and Urban Ward Act of 1976. In this study, Musoma Urban district was identified as a typical urban area, all district headquarters were assigned as urban areas, and all other areas outside of district headquarters were classified as rural areas.

### Data collection

Two methodologies were used in this study: 1) Extensive investigative interviews to determine rabies exposure incidence in the study areas, conducted in the three districts of Serengeti, Ulanga and Kilombero; and 2) questionnaires administered in all 4 study districts to determine health seeking behaviour, associated costs and coping strategies.

We compiled animal bite records and case reports from veterinary and livestock offices and details of patients reporting with animal-bite injuries from hospitals, clinics and dispensaries within the focal districts of Kilombero, Ulanga and Serengeti. These records were used to initiate extensive investigative interviews to determine networks of transmission; we actively searched and traced all identified sources of infection (i.e. biting animals) and subsequent cases of onward transmission/bite exposure, as previously described [6]. During the visits to households of bite victims, we collected information about the age, gender, and livelihood of bite victims, the severity of the bite, and the circumstances of the bite. Information was recorded on the attacking animal such as aggressive or abnormal behavior, drooling/salivation, vocalization, roaming, listlessness, or paralysis, and its subsequent fate. For known biting animals, information was collected from their owner (if identified) on how the animal may have become infected and whether it had any previous history of vaccination.

The aforementioned clinical signs were used to evaluate whether the attacking animal could be clinically categorized as rabid, based on the criteria of the ‘six-step’ method [29]. The ‘six step’ method is a diagnostic method based on epidemiological (history of exposure) and clinical criteria. Such criteria were previously reported to be accurate for 75% of specimens submitted for laboratory confirmation [30]. Whenever possible, brain stem samples from carcasses of animals that caused bite injuries were collected using the ‘straw’ technique as recommended by the World Health Organisation for laboratory confirmation [31], [32]. Universal Transverse Mercator coordinates were collected using a handheld Global Positioning System for each health facility and household visited and used to estimate an overall straight-line distance travelled by bite patients whilst seeking PEP.

A structured open-ended questionnaire was also administered to a subset of rabid animal bite victims and the families of rabies victims (n = 415, including 18 rabies death cases) bitten between January 2006 to December 2008. We collected information on demographic characteristics of the victims, costs related to the bite and seeking medical attention and coping strategies of bitten individuals and their families. We recorded the number of health facilities visited by the victim whilst seeking PEP, the number of days spent at each health facility, the amount of money paid for medical services, travel and other costs and how these funds were raised. When patients were escorted by an adult family member/guardian during the PEP seeking process, related costs were also captured. In order to validate costs incurred, heads of households were asked to produce receipts and in the absence of receipts, market prices or local fares were used to estimate the costs. Respondents were also asked to mention how much time both the patient and carer spent in seeking PEP. However, the questionnaire did not capture losses due to the premature death of a household member due to rabies or of being absent from school for school children.

Human demographic data for the study were drawn from the Tanzanian national population and housing census of 2002 [28]. The estimated population growth rates for each district according to the 2002 census were used to project the population size of the study areas for 2006–8. The total numbers of traced suspect bite and rabies death cases in the study areas for the period of January 2006–December 2008, together with the projected demographic data were used to estimate the annual incidence of rabies exposures and of human rabies deaths per 100,000 persons.

### Economic costs

For this analysis, the direct medical costs included the costs of biologicals (only rabies vaccines, since RIG was not administered in the study areas) and the costs associated with wound care, such as antibiotics, tetanus immunizations and disinfection. The price year was 2010. The indirect costs included out-of-pocket expenses for patients, such as transport costs to and from health centres and hospitals, accommodation and other costs for communication and subsistence while seeking PEP. Non-medical costs included productivity losses due to time spent seeking PEP. Time lost for both patients and escorts was valued in monetary terms according to projections of per capita daily income from the Tanzanian Household Budget Survey (HBS) of 2007 [33]. All medical and non-medical costs were expressed in terms of Tanzanian shillings (TZS), and converted to US dollars (US$) using the average annual exchange rate in 2010, which was 1 TZS to US$ 0.000687 [34]. The total number of PEP doses administered and patient visits made to health centres to receive PEP were derived from the questionnaires and validated from hospital records. We used the number of doses received to estimate patient compliance during PEP courses.

### Analysis

We calculated (i) average costs per suspect bite victim and (ii) average costs per dose. The average cost per suspect bite was defined as the average amount of cash spent by victims and caretakers following a bite, including costs of receiving PEP. Therefore suspect rabies bite victims including those who did not seek medical attention were included in this calculation. Average cost per dose was defined as the average amount of cash spent by patients and their carer(s) in receiving a single PEP dose. Therefore only patients who sought and successfully obtained at least one dose of PEP are included in this calculation. This was estimated by summing all cash costs spent on obtaining PEP and dividing by the total number of doses delivered. We also calculated the predicted average costs of PEP according to different regimens including intramuscular (IM) and intradermal (ID) administration and methods of subsidization that could be used in the future. These were calculated as the mean cost per dose plus annual income lost per dose, all multiplied by the number of clinic visits in the PEP regimen. These losses were converted into the percentage of annual income lost and equivalent days wages lost.

Chi-square tests were used to examine differences in sources of funds used to pay for PEP between victims from rural and urban areas and the proportion of victims that were escorted while seeking PEP. The Welch Two Sample t-test was used to determine the differences in average costs per suspect bite, numbers of hospitals visited before receiving PEP and delays in receiving PEP, between patients from rural and urban areas. Regressions were applied to assess factors affecting the costs of acquiring PEP, delays to obtaining PEP, probability of completing PEP (logistic regression) and probability of death following exposure to rabies (logistic regression). Explanatory variables investigated included direct medical costs, travel costs, accommodation costs, other costs, and loss of productivity costs, gender, residence (rural or urban), district and distance to PEP delivering hospitals. Every explanatory variable was tested to assess significance (at P<0.05) then a final multivariate regression model was developed using backward stepwise variable selection. All statistical analyses were implemented within the R statistical programming environment [35].

### Ethics statement

The study protocol was approved by the Medical Research Coordinating Committee of the National Institute for Medical Research of Tanzania, with approval number NIMR/HQ/R.8a/vol.IX/994 and the Institutional Review Board of the Ifakara Health Institute, including the use of oral consent for the collection of interview data. Written consent was not obtained as the study followed established procedures for collecting interview data in Tanzania without the collection of biological samples from humans. The study was cleared by the District Executive Director in every study district and Village Executive Officers were asked for permission prior to starting work in each village. Before administering questionnaires, participants were orally informed about the purpose of the study, the data to be collected and the freedom of their participation and their right to withdraw from the study at any time during the interview. Participants were then asked if they would wish to be interviewed. All respondents agreed verbally and the interviews were conducted.

## Results

### Incidence of rabies exposures and human rabies deaths

**Figure 2:**
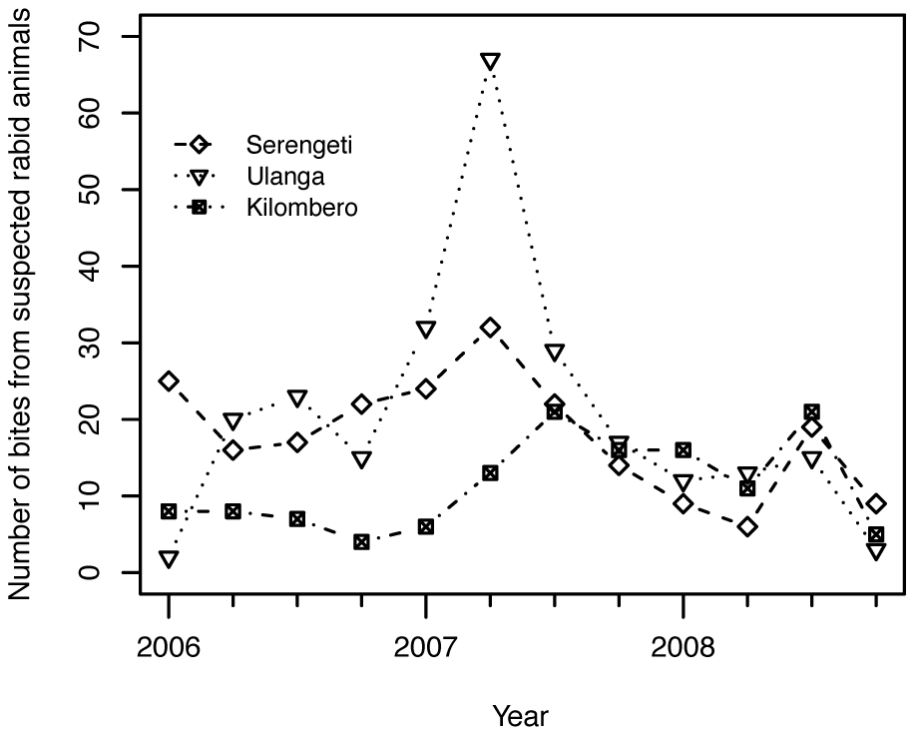
People bitten by suspected rabid animals traced per quarter from January 2006 to December 2008 in Ulanga, Kilombero and Serengeti districts, Tanzania.

Animal bite injuries were traced and investigated across three districts (Ulanga, Kilombero and Serengeti), from January 2006 to December 2009. Active searching revealed 599 animal bite injuries that met the case definition of being caused by suspect rabid animals as per criteria of the ‘six-step’ method: 136 in Kilombero district, 248 in Ulanga district and 215 in Serengeti district. Estimates of the annual incidence of bites from suspected rabid dogs per 100,000 persons, and annual incidence of human rabies deaths are summarised in Table 1. We were unable to trace all bite cases reported in hospital records; however, we suspect that using this methodology we captured the vast majority of rabies exposures including many instances where the bite victim did not report to hospital. The ages of suspect bite victims ranged from 1 to 90 years. The majority of suspect bite victims (51%) were children less than 15 years of age. The reported sources of bite exposure to humans were: domestic dogs (535 or 89%), others including humans (4 or 0.7%), livestock (1 or 0.2%), domestic cats (19 or 3%) and wild animals (32 or 5%). Reports of bites from wild animals included: jackals, honey badgers, genets, hyena, mongoose, wild pig and monkeys. The species were not determined due to potential ambiguities in identification. The peak of bites in Ulanga district was observed between July and September 2007 (Figure 2), when there was an outbreak of rabies in which 64 people were bitten by suspect rabid animals.

**Table 1:** Average annual incidence of suspect rabid animal bite injuries and human rabies deaths.

District	Human population*	Number of bites†			Number of deaths†			Average annual incidence/100,000	
		2006	2007	2008	2006	2007	2008	bites	deaths
Ulanga	193280	60	145	43	2	13	1	37.1	2.4
Kilombero	321611	27	56	53	3	2	5	11.3	0.8
Serengeti	176057	80	92	43	3	5	1	33.5	1.4

* According to the 2002 census;

† numbers of traced cases.

### Costs associated with receiving PEP

Structured open-ended questionnaires were administered to 415 bite victims bitten by suspect rabid animals between January 2006 and December 2008; 97 in Kilombero, 141 in Ulanga, 152 in Serengeti and 25 in Musoma Urban that were identified from hospital records. Questionnaires were not administered to all cases traced through active searching because of time constraints during fieldwork. Only 12% of these bite victims received PEP free of charge on a first-come first-served basis, the reminder had to pay for PEP, with rural bite victims paying around $12 per PEP dose and urban bite victims paying $8 per dose (Table 2). Direct medical costs per suspect bite victim ranged from zero for those who either received free medical services or did not seek medical attention, to more than US$250 for complicated bites, which involved surgical operations. There was no significant difference in productive days lost by bite victims from rural or urban areas, due to seeking health care following exposure (10.52 days in rural areas and 10.98 days in urban, p = 0.18). The average time lost per dose was very similar for rural and urban victims (5.3 days for rural victims and 5.2 days for urban victims) because the frequent shortages of PEP in district hospitals meant that patients living in district town centres (urban areas) also sometimes had to travel to major cities. The monetary value for time lost per suspect bite was equivalent to US$7.22 in rural areas whereas in urban areas it was estimated to be equivalent to US$17.03. The costs associated with receiving PEP including travel, accommodation and other costs are summarised in Table 2.

**Table 2:** Average cost per suspect bite and per PEP vaccination dose (in US$).

Components	Average cost per suspect bite (standard error)		Estimated cost per dose	
	Rural	Urban	Rural	Urban
Direct medical costs	23.85 (1.32)	17.32 (2.33)	12.01	8.19
Travel costs	9.51 (0.77)	4.21 (0.75)	4.79	1.99
Accommodation cost	2.20 (0.43)	4.18 (0.94)	1.11	1.98
Other costs	2.00 (0.54)	0.51 (0.27)	1.00	0.24
Lost income[days lost from work]	7.22[10.52 days]	17.03[10.94 days]	3.63	8.06
Total costs	44.78(1.74)	43.25(2.82)	22.54	20.46

Average cost per suspect bite is calculated based on all bite victims, whereas estimated cost per dose refers only to patients who enrolled in PEP.

### Hospital presentation and PEP administration

**Figure 3:**
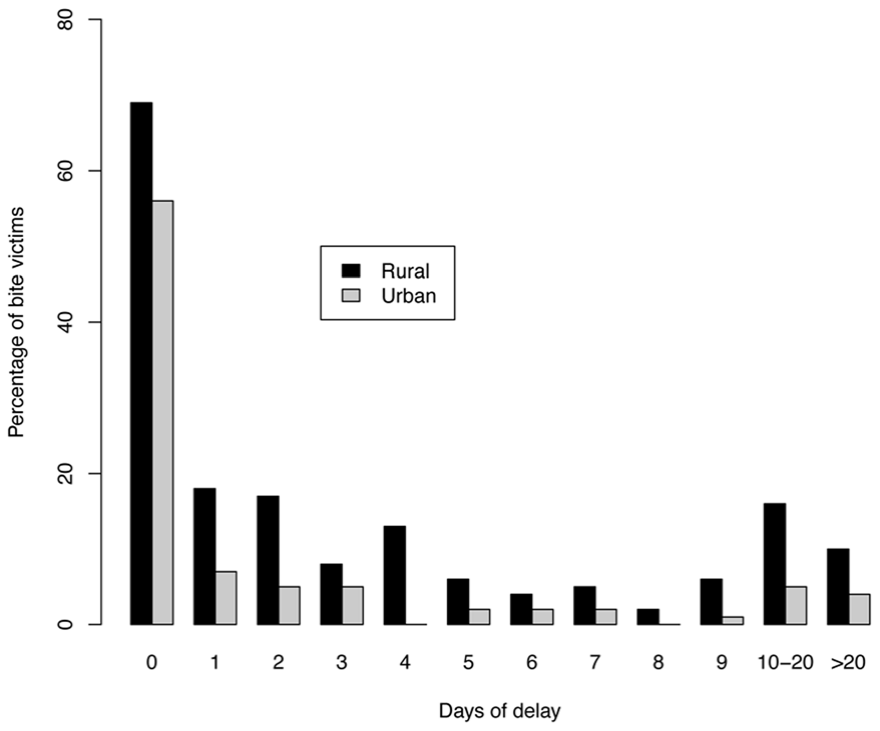
Distribution of delays before bite victims received their first dose of PEP.

Ninety-four percent (391/415) of these suspect bite victims reported to health facilities for PEP. About half of these bite victims reported, immediately after being bitten by animals but there was considerable variance in delays before receiving the first PEP dose between rural and urban bite victims. Bite victims in rural areas took longer, on average, to receive PEP than those in urban areas (Figure 3, mean 5.57 days, 95% CI 4.25–6.9 days in rural areas versus mean 3.64 days, 95% CI 2.09–5.19 days, in urban areas, χ^2^ = 10.91, df = 3, p = 0.01). Of 272 bite victims who lived far from district hospitals (more than 10 kilometres), only 39% received PEP within 7 days after exposure whereas, 64% of those who lived close to district hospitals received PEP within 7 days after exposure (χ^2^ = 14.59, df = 3, p = 0.002). Bite victims from urban areas where the district hospital was located in their home town did not incur many indirect costs compared with patients from rural areas who had to travel further to these hospitals. If PEP was not in stock at district hospitals, victims had to either wait for hospitals to procure PEP or travel elsewhere to receive PEP. We found that 40% (156/391) of patients attended multiple heath facilities (maximum of five heath facilities) for PEP. Of these 85% (132/156) were patients from rural areas (χ^2^ = 32.11, df = 2, p<0.0001).

A total of 841 doses were delivered to suspect rabid bite victims for the period between January 2006 and December 2008. Generally, patients were advised to get 3 doses of PEP intramuscularly on day 0, 7 and 28 according to Tanzanian health authorities, a regimen that is not recommended by WHO. On average suspect bite victims received 2.82 doses of PEP (95% CI 2.75 to 2.90 doses, Table 3). Almost 30% of suspect bite victims, who required PEP, did not receive any doses.

**Table 3:** Average distance to hospitals and the proportion of suspect bite victims to receive doses of PEP during 2006–2008.

District	Average distance to district hospital in kms (Std. Dev.)	Average distance to regional hospital in kms (Std. Dev.)	Suspect bite victims requiring PEP (n)	Suspect bite victims who got at least one PEP dose (%)	Suspect bite victims who got at least one PEP dose for free (%)	Number of PEP doses delivered	Average number of doses received by bite victims¥
Ulanga	20.68 (17.54)	241.24 (13.75)	141	96 (68%)	40 (28%)	259	2.70
Kilombero	23.91 (32.82)	193.01 (36.63)	97	81(84%)	8 (8%)	235	2.90
Serengeti	27.59 (15.92)	82.43 (18.19)	152	102 (67%)	2 (1%)	288	2.82
Musoma Urban	4.12 (1.36)	4.12 (1.36)	25	19 (76%)	0 (0%)	59	3.11
Overall rural	32.19 (20.64)	156.02 (80.72)	284	201(71%)	22 (8%)	564	2.81
Overall urban	2.97 (2.28)	160.74 (83.90)	131	97 (74%)	28 (21%)	277	2.86
Overall	22.96 (21.86)	157.51 (81.67)	415	298 (72%)	50 (12%)	841	2.82

¥based on persons that received one or more doses.

### Coping strategies for receiving PEP

**Figure 4:**
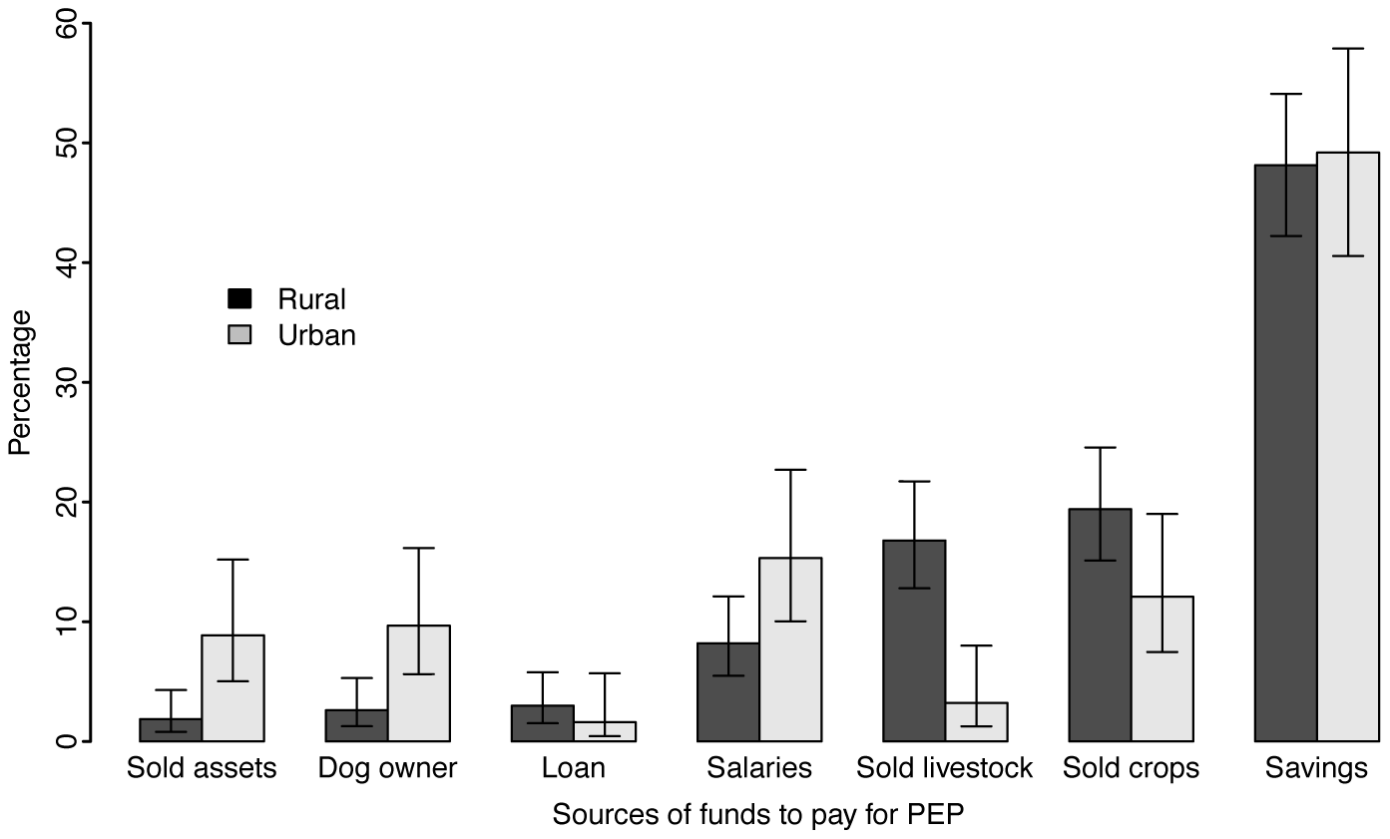
Sources of funds used by bite victims from rural and urban areas to pay for PEP.

The majority (73%) of suspect rabies bite victims were escorted by an adult family member to hospital. There was no significant difference in whether bite victims from rural versus urban areas were escorted to hospital (χ^2^ = 0.71, df = 1, p = 0.40). Families adopted various coping strategies to meet the costs of obtaining PEP. Families that had no savings had to transform their assets into cash. Poor rural families with little or no assets often were unable to afford PEP and experienced financial hardship while raising funds for PEP. We found that residence (rural or urban) had a significant impact on the source from which households obtained funds to pay for PEP (χ^2^ = 38.80, p<0.0001). Patients from urban areas were more likely to use money from their salaries or sell their assets whereas patients from rural areas either obtained funds from selling crops or selling livestock (Figure 4). Poor rural farmers were worst affected because they depend solely on one source of income (agricultural produce).

Despite the importance of PEP for saving the lives of bite victims, we found that shortages of PEP were common at the district hospitals. These shortages together with the expense of PEP created financial difficulties for many poor individuals, particularly those living in rural areas who had to raise money to pay for PEP, transport costs to reach urban areas and subsistence while receiving PEP. The implications of these costs associated with receiving PEP included delays in receiving PEP as summarised in figure 4, poor compliance with PEP regimens and human rabies deaths for those who did not obtain PEP.

**Figure 5:**
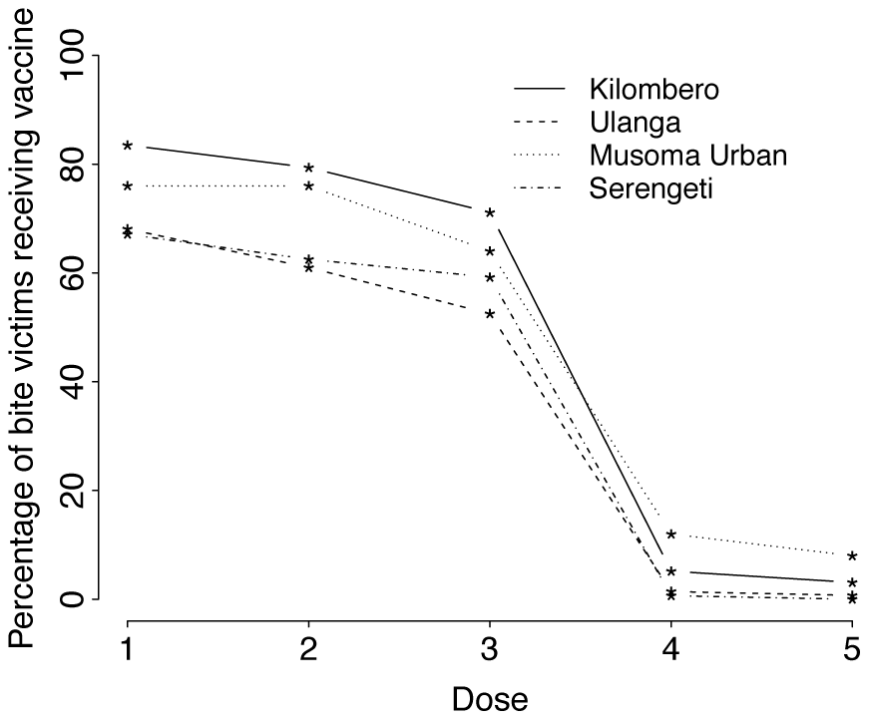
The probability of victims of bites by suspect rabid animals receiving and completing PEP vaccine doses. The situation for bite victims in Kilombero and Ulanga may not be representative as St Francis Mission Hospital in Kilombero received donated vaccine through Ifakara Health Institute as a result of the outbreak, therefore the population in the Kilombero valley is likely better served than in some other rural parts of Tanzania.

Just over 72% (298/415) of suspect bite victims received at least one dose of PEP. Of those that received their first dose, only 67% returned for a second dose, i.e. 5% of patients dropped out after a single dose. There was a further drop out of 16% after the second dose and very few patients received the last two doses (Figure 5). Patients from rural areas reported that they did not complete PEP because they were: i) unable to afford the vaccine, 54%; ii) vaccine was not available at the hospital, 32%; iii) the wound had already healed, 10%; iv) they were advised by the medical officer to discontinue PEP, 3% or v) they ignored advice to complete PEP, 1%. Reasons cited by urban patients were: i) lack of vaccine at the hospital, 64%; ii) unaffordable costs of vaccine, 24%; iii) advice from medical officers to discontinue PEP, 8%; and iv) because the wound had healed, 4%.

Costs were a significant obstacle for bite victims to both obtain and complete PEP (p<0.05). We therefore calculated the predicted costs of completing PEP under different regimens that are recommended by WHO, assuming PEP is provided free-of-charge, as well as being made available at local health centres and not just at district hospitals therefore minimizing travel costs. The costs of completing PEP under these scenarios are presented in Table 4.

**Table 4:** Average costs of PEP according to different schedules and methods of subsidization in relation to income.

Cost scenario		Cost of PEP (in US$)		Percentage of annual income (equivalent productive days)	
		Rural	Urban	Rural	Urban
5 dose Essen regimen	*No subsidies*	112.75	102.28	45.02 (164.34)	18.07 (149.07)
	*100% subsidised* §	52.70	61.31	21.04 (76.81)	10.83 (89.36)
	*100% subsidised and decentralised* ‡	23.20	41.48	9.26 (33.81)	7.33 (60.45)
Reduced 4 dose Essen regimenς	*No subsidies*	90.20	81.82	36.02 (131.47)	14.46 (119.25)
	*100% subsidised* §	42.16	49.05	16.84 (61.45)	8.67 (71.49)
	*100% subsidised and decentralised* ‡	18.56	33.18	7.41 (27.05)	5.86 (48.36)
Tanzanian 3 dose regimen	*No subsidies*	67.65	67.65	27.01 (98.60)	11.95 (98.50)
	*100% subsidised* §	31.62	36.79	12.63 (46.09)	6.50 (53.62)
	*100% subsidised and decentralised* ‡	13.92	24.89	5.56 (20.29)	4.40 (36.27)

The projected average per capita daily income for 2010 was USD 0.68 in rural areas and USD 1.55 in urban areas. PEP costs were calculated as: ((Cost per dose+(days lost per dose * daily income))*clinic visits;

§: Scenario with government providing PEP to bite patient free-of-charge;

‡*:* Scenario with government providing PEP to bite patient free-of-charge from local health facilities (i.e. not only at district hospitals).

ςThis regimen requires 4 hospital visits and therefore is equivalent in travel costs to the reduced Thai Red Cross ID regimen that uses less vaccine (0.1 ml/injection).

We found that 28% (117/415) of bite victims did not receive PEP of any kind, of them 18/117 (15%) developed rabies, whereas none of those who received a single dose of PEP developed rabies. Most of the deaths occurred in households dependent on subsistence farming (89%), involved children less than 16 years of age (78%) and occurred in areas far from hospital (78% of deaths occurred in areas more than 10 kilometers away from nearest district hospital and 89% further than 60 kilometers from the regional hospital). In a multivariate analysis, distance to the district hospital and direct medical costs were the only significant predictors of human rabies deaths (p<0.05).

## Discussion

The present study confirms that rabies remains a serious public health and economic problem in developing countries where canine rabies is endemic. Bites from suspect rabid animals impose a substantial financial burden on affected households, especially for poor rural bite victims who suffer excessively high costs in obtaining PEP compared to those from urban areas, and are less likely to receive vaccine. PEP is vital for human rabies prevention and requires repeat visits to hospital to complete a full course. Rural bite victims often live far from major health facilities in urban areas, where PEP can be obtained; however, even in these areas the availability of PEP is not guaranteed. In remote areas, travel to a regional hospital where PEP shortages are less frequent, can take more than 18 hours and is expensive. Moreover, costs were almost double for patients that needed to be escorted by a family member/adult, with rural bite victims incurring disproportionately higher indirect costs and a higher risk of developing rabies and dying from the disease. Delays to receiving PEP, due to hospital shortages and time spent raising money or waiting for district hospitals to procure PEP increased the risk of developing this fatal disease. The need to repeatedly visit hospital to complete PEP may deter poor people living in remote rural areas from obtaining and completing PEP, which may explain why the risk of rabies was higher for those living further from major hospitals. More generally, the distance to major hospitals seems to be an obstacle for people living in rural areas in Africa. A study in Ivory Coast reported that over 75% of patients who discontinued of PEP were from outside the capital city, Abidjan [36]. In summary, we have shown that major inequalities in health care, and access to and affordability of PEP for bite victims exist in Tanzania and have demonstrated the importance of evaluating health-seeking behavior in local settings.

Adoption of a reduced 4-dose regimen such as the Essen 4-dose or the Zagreb regimen could reduce the number of hospital visits that patients need to make to complete their PEP course, in comparison to the 5-dose Essen regimen currently recommended by WHO. In several Asian countries ID regimens are being used that can reduce the total volume of vaccine required by up to 80%, and also only require 4 hospital visits [37]. However, even with the adoption of reduced 4-dose regimens (IM or ID) indirect costs alone would still be between US$ 40–50. This is equivalent to almost 20% of the average annual income in rural Tanzania, and is therefore unaffordable for many rural Tanzanians. For patients bitten by healthy animals, follow up of the biting animal (vaccination status and 10-day observation period) could be used to discontinue PEP and reduce costs. However, our study shows that clinicians would require training to provide this advice as on some occasions discontinuation of PEP was given when the biting animal was suspect for rabies. It is not clear why the Tanzanian government generally administers a 3-dose regimen, but we did not find any deaths as a result of this unconventional regimen, which suggests that further research into reduced dose regimens is warranted.

In Tanzania, although the government aims to provide PEP free-of-charge, budgets allocated for this are often insufficient, resulting in shortages. We found that PEP was provided free-of-charge to less than 20% of bite victims, whilst the reminder had to pay, incurring costs that were equivalent to around two months income. If patients fully adhered to recommended regimens these costs would be considerably higher. We show that if PEP was fully subsidized and reduced dose regimens were instead adopted, costs to patients would be substantially reduced. Therefore, it is important for governments to consider strategies that increase accessibility and affordability of PEP to the rural poor who are at the most risk from the disease. This would assist poor families with few assets or little means to pay, especially poor farmers who depend on selling farm outputs to obtain money for PEP.

In comparison to the literature, our study suggests that costs of seeking PEP are substantially underestimated. A previous study estimated that a full course of PEP (direct and indirect costs, excluding those of any patient escorts) to be US$ 39.57 in Africa including 1% of patients receiving RIG [7]. Our results showed that for a full course of PEP excluding RIG, a rural Tanzanian patient would have to pay more than US$100 following a 5-dose regimen, hence we recommend re-evaluation of the burden of rabies in Africa. Furthermore, our study highlighted inequalities such as travel costs for rural patients being more than double than those from urban areas. Our study did not include annual expenditure for rabies control in dogs (previously estimated at $9.7 million) or the long-term impacts of the death of a household member due to rabies, absence from school by schoolchildren and psychological impacts of exposure and death from the disease. These additional factors are likely to considerably increase the longer-term economic impacts from rabies exposure beyond what has been estimated here.

One of the most effective methods to reduce expenditure on PEP is for veterinary services to invest in mass dog vaccination. Studies in northwest Tanzania have demonstrated the impact of mass dog vaccination on reducing animal-bite injuries and demand for PEP [38], [39]. Strategies to prevent human rabies should therefore also include mass vaccination of domestic dog populations, which maintain the disease. High levels of mass dog vaccination coverage in Africa can be achieved at a relatively low cost, estimated at less than US$2 per dog [39], [40] which make this a highly effective way to control rabies in the medium- to long-term [41]. If major investments would be made to control rabies in dogs, the majority of African countries could likely afford to subsidize PEP. However, this requires the adoption of a one health approach, involving collaboration and sharing of information between public health and veterinary services for effective rabies control and prevention [42].

Recently we have seen an increased effort by the international community to improve the health of the world's poor. Attention has been focused on the relationship between health and poverty, particularly in relation to the Millennium Development Goals (MDGs) [43]. One of the major goals of the MDGs is a 50% reduction in the number of people living in absolute poverty by 2015. Diseases such as malaria, HIV/AIDS and TB have featured prominently in terms of attracting funding for achieving these goals because they have been prioritized by the international health community and donor agencies due to the high number of fatalities they cause in Africa each year. Rabies is severely neglected and its control is overlooked by authorities. However, this study demonstrated that the burden of rabies for poor households is substantial and calls for national and global attention. Integrating rabies control into Tanzania's National Strategy for Growth and Reduction of Poverty (NSGRP) would contribute to meeting the MDGs, particularly of eradicating extreme poverty (MDG 1) and combating HIV/AIDS, malaria, and other diseases (MDG 6). Specifically our data shows that rabies imposes a disproportionate financial hardship and high risk of dying of rabies to rural poor families because of the need to access costly PEP promptly and highlights the need to re-evaluate the burden of rabies in Africa.
